# Post-myocardial Infarction Ventricular Septal Defect: A Case Report of a Rare but Devastating Complication

**DOI:** 10.7759/cureus.79415

**Published:** 2025-02-21

**Authors:** Bola Habeb, Lillian Short, Nur Mando, Erica S Thomson, Rohit Amin

**Affiliations:** 1 Internal Medicine, University of Florida College of Medicine, Ascension Sacred Heart, Pensacola, USA; 2 Cardiology, University of Florida College of Medicine, Ascension Sacred Heart, Pensacola, USA

**Keywords:** heart failure post-stemi, holosystolic murmur, ischemic vsd repair, left-to-right shunt, post-mi complications, post-myocardial infarction ventricular septal rupture, post-myocardial infarction vsd, surgical septal repair

## Abstract

Post-myocardial infarction ventricular septal defect (PMI-VSD) is a rare but serious complication of acute myocardial infarction, characterized by a rupture in the interventricular septum. Patients typically present with symptoms of cardiogenic shock, a new systolic murmur, and heart failure. Diagnosis is confirmed through echocardiography with Doppler imaging. Management involves hemodynamic stabilization and definitive repair via surgical or percutaneous approaches. Despite advances in treatment, PMI-VSD is associated with high mortality, highlighting the importance of early detection and prompt intervention.

## Introduction

Heart disease is the first leading cause of death in the United States. One person dies every 33 seconds from cardiovascular disease. Myocardial infarction is one of the most serious and fatal causes of heart disease. In recent decades, advancements in pharmacological treatments, catheter-based interventions, and surgical reperfusion techniques have improved the outcomes for patients with acute myocardial infarctions. Patients with large transmural inferior and anterior infarcts, those who were left untreated, or those who underwent delayed interventions remain at a higher risk for post-myocardial infarction mechanical complications [1,2]. Ventricular septal defect (VSD) is a rare but lethal complication of myocardial infarction with an incidence rate of 0.17-0.44% [3]. Risk factors include female sex, older age, chronic kidney disease, delayed reperfusion, single vessel disease, and poor septal collateral flow [3,4]. The condition leads to the formation of a left-to-right shunt between the ventricles, which can result in rapid hemodynamic deterioration, acute heart failure, and cardiogenic shock. Managing infarct-related mechanical complications is intricate, necessitating a multidisciplinary collaborative approach for timely recognition, hemodynamic stabilization, and early intervention to improve outcomes. Herein, we underscore the critical importance of early identification of VSD as a rare mechanical complication of myocardial infarction. We also emphasize the urgency of prompt intervention to facilitate effective management and reduce mortality.

## Case presentation

A 79-year-old female with a medical history significant for hypertension, non-insulin-dependent type II diabetes mellitus, and coronary artery disease was admitted to an outside facility for the management of an anterior ST-elevation myocardial infarction (STEMI). Initial laboratory evaluation showed an elevated troponin level with no other significant abnormalities (Table 1).

**Table 1:** Laboratory data obtained on admission BUN: blood urea nitrogen, BNP: brain natriuretic peptide, AST: aspartate aminotransferase, ALT: alanine aminotransferase

Parameters	Patient's values on admission	Reference range (adult)
Hemoglobin (g/dL)	15.5	12.0-15.5
Hematocrit (%)	44.2	34.9-44.5
White cell count (per mm3)	12300	3,500-10,500
Platelet count (per mm3)	256,000	150,000-450,000
Sodium (mEq/dL)	135	135-145
Potassium (mEq/dL)	4.4	3.5-5.1
Bicarbonate (mEq/dL)	21	22-29
BUN (mg/dL)	24	12-21
Troponin (ng/mL)	11.75	0.010-0.013
BNP (pg/mL)	285.4	10-100
Lactate (mmol/L)	1.5	0.9-1.7
AST (units/L)	73	12-31
ALT (units/L)	26	9-29
Magnesium (mg/dL)	1.8	1.6-2.6
Phosphorus (mg/dL)	2.8	2.3-4.7

**Figure 1:**
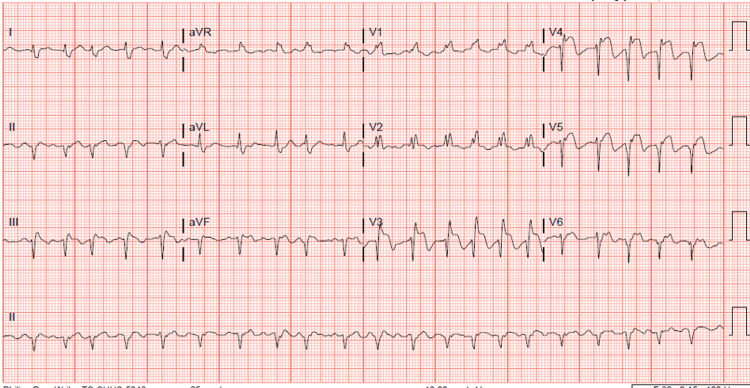
A 12-lead ECG demonstrating ST-segment elevations in the anterolateral leads, accompanied by Q waves, as well as ST-segment elevations in leads II, III, and aVF ECG: electrocardiogram

A 12-lead electrocardiogram (ECG) demonstrates ST-segment elevations in the anterolateral leads, accompanied by Q waves, and ST-segment elevations in leads II, III, and aVF (Figure 1).

A transthoracic echocardiogram (TTE) showed normal left ventricle size, apical akinesia, and mildly depressed function, with an estimated ejection fraction EF of 45% (Video 1).

**Video 1:** TTE apical 4 (A-4) view showing mildly reduced systolic function (EF 45%) TTE: transthoracic echocardiogram, EF: ejection fraction

The patient underwent left heart catheterization, which revealed a diffusely diseased left anterior descending artery with a 100% occlusion in the distal segment. There was TIMI 0 flow beyond the occlusion in a vessel measuring approximately 1.5 mm in diameter. Considering the patient’s coronary anatomy, the time elapsed since symptom onset, and the absence of ongoing symptoms, a decision was made to defer percutaneous coronary intervention (PCI). The patient was discharged with recommendations to continue anti-ischemic medical therapy, including aspirin 81 mg daily, metoprolol succinate 25 mg daily, lisinopril 20 mg daily, and atorvastatin 40 mg daily.

Two weeks later, the patient was admitted to our facility with symptoms of left ventricular failure (i.e., dyspnea, orthopnea, and paroxysmal nocturnal dyspnea) and a new 4/6 holosystolic murmur best heard at the left lower sternal border and propagated to the back. Laboratory data on the readmission is demonstrated in (Table 2).

**Table 2:** Laboratory data obtained on readmission BUN: blood urea nitrogen, BNP: brain natriuretic peptide, AST: aspartate aminotransferase, ALT: alanine aminotransferase

Parameters	Patient's values on admission	Reference range (adults)
Hemoglobin (g/dL)	13.8	12.0-15.5
Hematocrit (%)	38.9	34.9-44.5
White cell count (per mm3)	15700	3,500-10,500
Platelet count (per mm3)	199,000	150,000-450,000
Sodium (mEq/dL)	138	135-145
Potassium (mEq/dL)	4.8	3.5-5.1
Bicarbonate (mEq/dL)	24	22-29
BUN (mg/dL)	30	12-21
Troponin (ng/mL)	0.5	0.010-0.013
BNP (pg/mL)	975.7	10-100
Lactate (mmol/L)	1.8	0.9-1.7
AST (units/L)	43	12-31
ALT (units/L)	28	9-29
Magnesium (mg/dL)	2.2	1.6-2.6
Phosphorus (mg/dL)	3.8	2.3-4.7

**Figure 2:**
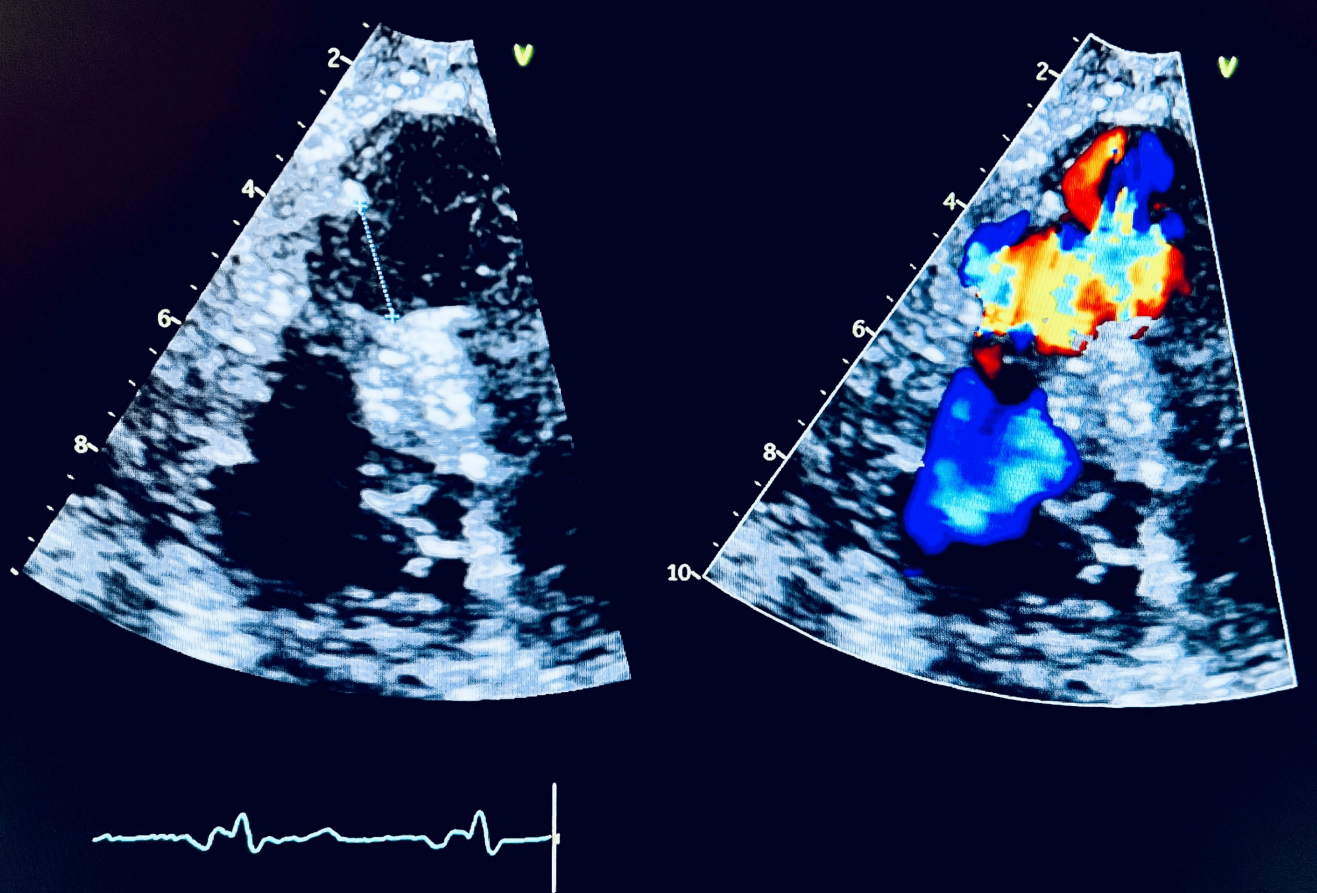
Still image from the patient’s TTE showing a large VSD with left-to-right shunting toward the apex TTE: transthoracic echocardiogram, VSD: ventricular septal defect

A repeat TTE showed normal left ventricle size with hyperdynamic systolic function, normal right ventricle size with depressed systolic function, and a large apical muscular VSD measuring about 2.2 cm with a left-to-right shunt (Figure 2, Video 2).

**Video 2:** TTE apical 4 (A-4) view showing a large apical muscular VSD TTE: transthoracic echocardiogram, VSD: ventricular septal defect

Cardiothoracic surgery was consulted, and the patient underwent preoperative intra-aortic balloon pump (IABP) placement, followed by a successful VSD repair using a bovine patch. Postoperatively, the patient was successfully weaned off mechanical ventilation and circulatory support, experiencing a smooth recovery with symptomatic improvement and resolution of the murmur. At a six-month follow-up, the patient reported a complete resolution of symptoms and a return to baseline functional status. A repeat TTE demonstrated no evidence of a left-to-right shunt (Video 3).

**Video 3:** TTE apical 4 (A-4) view at the six-month follow-up showing the successful resolution of the VSD with no evidence of a left-to-right shunt TTE: transthoracic echocardiogram, VSD: ventricular septal defect

## Discussion

PMI-VSD is a rare but life-threatening complication, occurring in ~0.3% of myocardial infarction cases compared with 1-2% before thrombolysis and reperfusion therapy was introduced [3,5]. Ventricular septal rupture (VSR) can occur acutely within 24 hours post-myocardial infarction secondary to dissecting intramural hematoma or sub-acutely within three to seven days as a direct consequence of myocardial ischemia, coagulative necrosis, neutrophilic and macrophage infiltration to remove the necrotic tissue, and subsequent tissue weakness [5]. VSR is most commonly associated with anterior and inferior STEMI secondary to complete coronary occlusion with the absence of collateral vessels.

Symptoms and signs vary according to the VSD size and the presentation time, ranging from mild exertional dyspnea to cardiogenic shock. A new loud holosystolic murmur on physical examination may be heard at the left sternal border, often radiating across the chest.

Early diagnosis of PMI-VSD is essential for survival. TTE is the diagnostic modality of choice, providing a clear visualization of the septal defect and the shunting of blood from the left to the right ventricle. Doppler echocardiography can quantify the severity of the shunt, helping guide management decisions. Additionally, right heart catheterization can assess the magnitude of the left-to-right shunt by measuring oxygen saturation differences between the chambers. In some cases, cardiac MRI or CT scans may be used to further assess myocardial viability and defect size.

The management of PMI-VSD requires a multidisciplinary approach, including cardiologists, cardiothoracic surgeons, and intensive care specialists. Initial stabilization of the patient’s hemodynamics is crucial; this may involve inotropes to improve cardiac output and vasodilators to reduce afterload. Mechanical support with an IABP is often used to decrease left ventricular afterload and reduce the shunt volume, temporarily improving circulation until surgical repair can be performed. However challenging due to tissue friability, surgical closure with a patch is still the standard treatment. It should be considered as soon as the patient stabilizes, as delays increase the risk of hemodynamic collapse.

Percutaneous (trans-catheter) closure is a less invasive alternative to surgery in select cases, particularly in smaller or more stable VSDs; however, it is not universally applicable, especially for larger defects with significant tissue necrosis. A systematic review studying the differences between management options found no significant difference in mortality among patients with an early transcatheter approach versus the surgical closure group [6].

Despite advances in treatment, the prognosis of patients with PMI-VSD remains guarded. VSD has a high mortality rate of 45% in patients undergoing surgical repair compared to 90% if left untreated [5]. Long-term survival after repair is largely dependent on the extent of the initial myocardial damage and the success of the surgical intervention. Close follow-up with echocardiography is essential to monitor for residual shunts or the development of heart failure.

## Conclusions

VSD is a rare but devastating complication following myocardial infarction, particularly in cases of anterior and inferior STEMI. Early recognition, hemodynamic stabilization, and timely surgical intervention are critical in optimizing patient outcomes. While percutaneous closure presents a novel option in select cases, surgical repair remains the gold standard. Advances in reperfusion techniques, such as timely PCI, have significantly reduced the incidence of PMI-VSD, highlighting the importance of early intervention in acute myocardial infarction management.

Ongoing research into novel therapeutic strategies and advancements in perioperative care is essential to overcoming the challenges associated with this condition. Prompt detection and rapid management remain pivotal in mitigating the substantial morbidity and mortality linked to PMI-VSD.
